# Upregulation of CEP55 Predicts Dismal Prognosis in Patients with Liver Cancer

**DOI:** 10.1155/2020/4139320

**Published:** 2020-04-12

**Authors:** Lingpeng Yang, Yang He, Zifei Zhang, Wentao Wang

**Affiliations:** ^1^Liver Surgery Department, Liver Transplantation Center, West China Hospital of Sichuan University, Chengdu, Sichuan Province, China; ^2^General Surgery Department, The Affiliated Hospital of Xizang Minzu University, Xianyang, Shaanxi Province, China

## Abstract

**Purpose:**

This study was performed to investigate the association of CEP55 expression with liver cancer and explore potential underlying mechanisms. *Materials and Methods*. Data obtained from The Cancer Genome Atlas (TCGA) was used to investigate CEP55 expression, its prognostic value, the potential mechanisms of its upregulation, CEP55-related pathways, and its biological functions in liver cancer. Data from Gene Expression Omnibus (GEO) and International Cancer Genome Consortium (ICGC) was used to validate survival analysis. The correlation between CEP55 and tumor-infiltrating immune cells (TIICs) in liver cancer was determined by using Tumor Immune Estimation Resource (TIMER).

**Results:**

CEP55 was significantly overexpressed in the liver tumor sample compared to the adjacent normal liver sample. High CEP55 expression was significantly associated with histological grade, advanced stages, histological type, high T classification, and survival status. High CEP55 expression was significantly related to dismal prognosis compared with low CEP55 expression, which was validated by the GSE54236 dataset and ICGC database. Meanwhile, CEP55 was identified as the risk factor to independently predict overall survival (OS) for patients with liver cancer upon multivariate analysis. Enrichment analysis indicated that cell cycle, DNA replication, pathways in cancer, mTOR signaling pathway, and VEGF signaling pathway were significantly enriched in the high CEP55 expression group. In addition, the CEP55 expression was significantly related to the infiltration level of B cells, CD4+ T cells, CD8+ T cells, macrophages, neutrophils, and dendritic cells in hepatocellular carcinoma (HCC). CEP55 methylation level was negatively correlated to its mRNA expression. And patients with CEP55 hypermethylation and low expression can achieve a better prognosis than those with CEP55 hypomethylation and high expression.

**Conclusion:**

CEP55 may serve as a candidate treatment target for it is a determinant of prognosis and immune infiltration in liver cancer patients. DNA hypomethylation might contribute to the overexpression of CEP55 in liver cancer.

## 1. Introduction

Liver cancer ranks the 6^th^ place in terms of global tumor incidence, and it is the 4^th^ leading cause of cancer-related deaths [1]. Hepatocellular carcinoma (HCC), one of the frequently seen primary liver tumors, occupies about 80% of liver cancers, followed by cholangiocarcinoma (CCA), accounting for approximately 15%. Worldwide, the highest liver cancer morbidity is reported in Asia and Africa. Approximately 75% of liver cancer occurs in Asia, and China accounts for more than half of the total cases in the world. Aflatoxin exposure and chronic hepatitis B virus (HBV) infection are two major risk factors reported across most high-incidence countries in Asia and Africa [2]. Although great progresses have been achieved in diagnosing and treating liver tumor over the past few decades, prognosis for liver tumor remains dismal. Liver cancer has become the second most fatal tumor after pancreatic cancer with a 5-year survival rate of 18% [3]. Finding reliable predictors and potential therapeutic targets involved in the occurrence and development of liver cancer is urgently needed.

Centrosome proteins have long been considered to be scaffold proteins that regulate mitotic spindle and microtubule tissue, so they are essential to the cell cycle process [4]. Centrosome protein 55 (CEP55), also referred as FLJ10540 and C10orf3, was initially recognized as a key component of abscission, which is the final stage of cytoplasmic division that is responsible for regulating the physical disjunction of two daughter cells [5]. CEP55 is located in the centrosome during the whole cell cycle, in the mitotic spindle during mitosis, and in the midbody during the process of cytokinesis [6]. Cytokinesis is strictly controlled in the process of cell division and requires the recruitment of multicomponent subunits to the midbody in a CEP55-dependent manner [4, 7, 8]. CEP55 has been identified both as a cancer testis antigen and a tumor-associated antigen [9, 10]. Cancer testis antigens are proteins normally expressed predominantly in the testes but which become more widely expressed in cancer [11]. Recently, some literature reported that CEP55 participated in promoting tumorigenesis and regulating the PI3K/AKT signaling pathway [12, 13]. Growing evidence indicates the association between CEP55 upregulation and the development and progression of a variety of malignant tumors, including breast tumors, gastric tumors, and lung tumors [13–15]. The knockdown of CEP55 can significantly inhibit the viability and proliferation of a tumor cell and even lead to tumor cell death [16, 17].

Though Li et al. [18] previously reported that the overexpression of CEP55 can result in poor prognosis of liver patients, their study is limited to HCC. In this study, we included both HCC and CCA patients from The Cancer Genome Atlas (TCGA) to investigate the prognostic significance of CEP55 expression and DNA methylation of CEP55 in liver cancer. The biological functions and signal pathways associated with CEP55 regulatory mechanism were also explored. In addition, we investigated the correlation between CEP55 and tumor-infiltrating immune cells (TIICs) in liver cancer using Tumor Immune Estimation Resource (TIMER).

## 2. Materials and Methods

### 2.1. Data Mining and Collection

**Figure 1:**
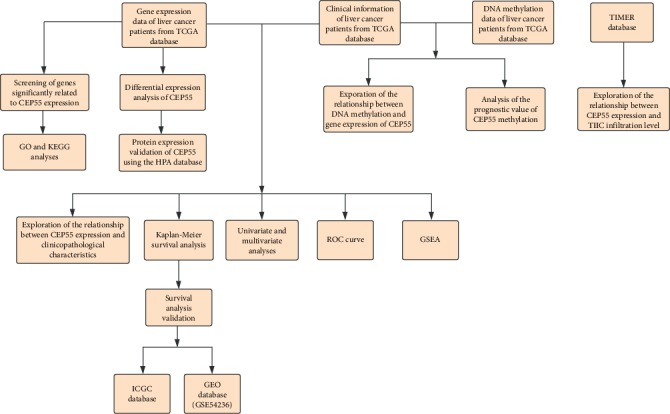
Flow chart of the study design.

A flow chart of our study design is showed in Figure 1. The gene expression profile data, DNA methylation data, and the clinical information of liver cancer patients had been collected from TCGA (https://cancergenome.nih.gov/). The clinical data included age, sex, survival time, survival status, clinical stage, histological grade, TNM classification, and histological type. Additionally, gene expression data of liver cancer patients with survival information was downloaded from the International Cancer Genome Consortium (ICGC) (http://icgc.org/). And GSE54236 dataset was obtained from the Gene Expression Omnibus (GEO) (https://www.ncbi.nlm.nih.gov/geo/). The expression levels of CEP55 in several common cancers were reviewed by using the TIMER database (https://cistrome.shinyapps.io/timer/). The CEP55 protein expression in normal liver samples and liver cancer samples was examined using immunohistochemistry (IHC) data from the Human Protein Atlas (HPA) database (http://www.proteinatlas.org/).

### 2.2. Functional Enrichment Analysis

Genes significantly related to CEP55 expression were extracted using the Pearson correlation analysis (∣*R*∣ ≥ 0.4, *p* < 0.001), and these genes were sent for Gene Ontology (GO) and Kyoto Encyclopedia of Genes and Genomes (KEGG) enrichment analyses using the clusterProfiler package (version 3.10.0). Gene terms with *p* value < 0.05 and false discovery rate (FDR) < 0.05 were considered significantly.

### 2.3. Gene Set Enrichment Analysis (GSEA)

GSEA had been carried out for determining whether an a priori defined set of genes showed significant differential expression between the high and low CEP55 expression groups and to identify the potential mechanisms of CEP55 expression in liver cancer prognosis. Each analysis was run 1000 times gene set permutation. CEP55 expression level had been utilized to be a phenotype label. Gene sets with normalized (NOM) *p* value < 0.05 and FDR < 0.05 were considered significantly enriched.

### 2.4. Immune Infiltrates Correlation via TIMER

Associations between CEP55 expression and TIIC infiltration levels were analyzed via TIMER platform, a web tool for gene-specific correlational analysis with TIICs [19]. TIICs included B cells, CD4+ T cells, CD8+ T cells, macrophages, neutrophils, and dendritic cells.

### 2.5. Analysis of DNA Methylation of CEP55

The relationship between CEP55 methylation and its mRNA expression was determined using the Pearson correlation analysis. Liver cancer patients were divided into the hypermethylation and hypomethylation groups according to the median CEP55 methylation level. The prognostic value of CEP55 methylation was evaluated by the Kaplan-Meier analysis.

### 2.6. Statistical Analysis

The R software 3.5.0 was utilized for statistical analysis. The expression of CEP55 in liver tumor samples and adjacent normal samples was compared using the Wilcoxon rank sum test. Patients with liver tumors were classified as the high or low expression group based on the median value of the CEP55 expression. The relationship between clinicopathological characteristics and CEP55 expression had been examined by logistic regression and Wilcoxon signed-rank test or Kruskal-Wallis test. Overall survival (OS) in the high CEP55 expression group was compared with that in the low CEP55 expression group by the Kaplan-Meier method. The *p* values were computed using a log-rank test. The receiver operating characteristics (ROC) curve was used to evaluate the prognostic value of CEP55 in 1, 3, and 5 years by using the survivalROC package. Univariate as well as multivariate Cox analysis was conducted for determining the relationships of CEP55 expression with OS as well as other clinical features (such as age, sex, clinical stage, histological grade, and TNM classification). A difference of *p* < 0.05 was deemed to be of statistical significance.

## 3. Results

### 3.1. Clinical Features for Liver Cancer Patients

The clinical data from 418 liver cancer patients (including age, sex, survival status, clinical stage, histological type, TNM classification, and histological grade) were downloaded from the TCGA database, as summarized in Table 1. Altogether, 272 male cases (65.07%) as well as 146 female cases (34.93%) had been included in this study, of whom 371 had HCC (90.19%) and 41 had CCA (9.81%). Upon completion of the follow-up, 271 cases survived (64.83%) and 147 patients died (35.17%). Stage I tumors were found in 194 patients (46.41%), stage II tumors in 98 patients (23.44%), stage III tumors in 90 patients (21.53%), and stage IV tumors in 12 patients (2.87%). According to the histological grade, 55 patients had G1 (13.16%), 180 patients had G2 (43.06%), 124 patients had G3 (29.67%), and 13 patients had G4 (3.11%) disease. Based on the T classification, 204 patients had T1 (48.80%), 107 patients had T2 (25.60%), 90 patients had T3 (21.53%), and 14 patients had T4 (3.35%) disease. Eight patients (1.91%) had lymph node metastasis, and eight patients (1.91%) had distant metastasis.

### 3.2. The mRNA and Protein Expressions of CEP55

**Figure 2:**
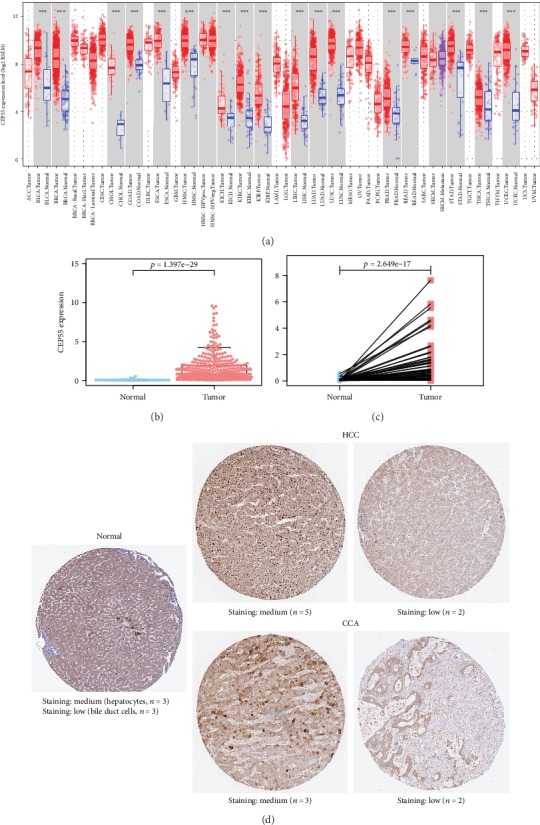
CEP55 expression both in the mRNA and protein levels. (a) Overview of CEP55 mRNA expression in different tumor tissues and adjacent normal tissues via the TIMER database (^∗∗∗^*p* < 0.001). (b) Comparison of CEP55 expression between liver cancer tissues and adjacent normal liver tissues. (c) Expression of CEP55 in 58 paired liver cancer tissues and adjacent normal tissues. (d) Representative IHC images of CEP55 in normal liver tissues and liver cancer tissues from the HPA database. TIMER: Tumor Immune Estimation Resource; HPA: Human Protein Atlas.

The CEP55 expressions were reviewed in different tumors via the TIMER database. Compared to normal tissues, CEP55 expression was extremely higher in most common tumor tissues, such as breast cancer, liver cancer, colorectal cancer, and gastric cancer (Figure 2(a)). CEP55 expression in liver tumor samples and adjacent normal samples was also compared using data directly obtained from TCGA, and the result demonstrated that CEP55 expression was markedly increased within liver cancer samples compared to normal liver samples (*p* = 1.397*e* − 29) (Figure 2(b)). Furthermore, the expression of CEP55 in 58 paired liver cancer samples and adjacent normal samples was analyzed, and our findings suggested a marked overexpression of CEP55 for liver tumor (*p* = 2.649*e* − 17) (Figure 2(c)). To further examine the protein expression of CEP55, we retrieved the IHC staining data from the HPA (Figure 2(d)). In three normal liver samples, all hepatocytes had medium CEP55 staining, while all bile duct cells had low CEP55 staining. In tumor samples, most of the HCC samples had medium (5/7) CEP55 staining, and most of the CCA samples similarly had medium (3/5) CEP55 staining. The results showed that CEP55 had a significantly higher protein expression in the CCA samples than in the normal liver samples. Although CEP55 had a significantly higher mRNA expression in the HCC samples than in the normal liver samples, there was no significant difference in the protein expression between the HCC samples and normal liver samples.

### 3.3. Association between CEP55 Expression and Clinicopathological Characteristics

**Figure 3:**
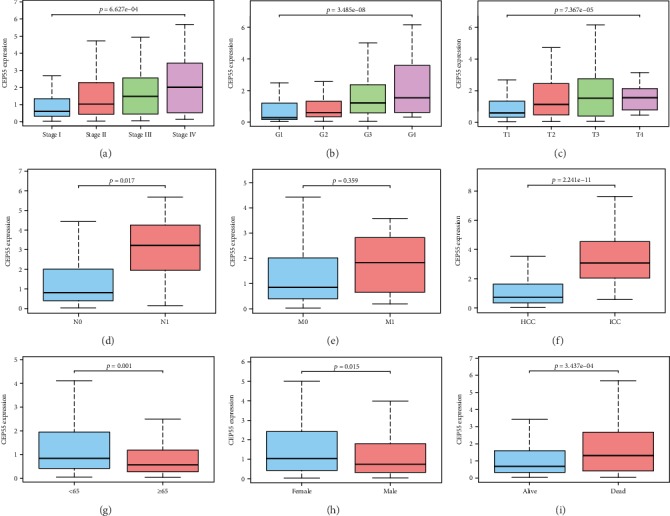
Association between CEP55 expression and clinicopathological characteristics. (a) Clinical stage. (b) Histological grade. (c) T classification. (d) N classification. (e) M classification. (f) Histological type. (g) Age. (h) Gender. (i) Survival status.

The CEP55 expression data and clinicopathological information from 418 patients with liver tumors were obtained from the TCGA database for analysis. The results are illustrated by box plots in Figures 3(a)–3(i). High CEP55 expression was significantly related to clinical stage (*p* = 6.627*e* − 4), histological grade (*p* = 3.485*e* − 8), T classification (*p* = 7.367*e* − 5), lymph node metastasis (*p* = 0.017), histological type (*p* = 2.241*e* − 11), age (*p* = 0.001), survival status (*p* = 3.437*e* − 4), and sex (*p* = 0.015). Univariate analysis using logistic regression indicated that high CEP55 expression (based on the median value of the CEP55 expression) was related to poor prognostic clinicopathological characteristics (Table 2). High CEP55 expression within liver tumors showed a significant association with high histological grade (OR = 3.06 for G1/G2 vs. G3/G4, *p* = 9.08*e* − 7), advanced stages (OR = 1.97 for I/II vs. III/IV, *p* = 0.005), histological type (OR = 18.13 for HCC vs. CCA, *p* = 8.43*e* − 5), high T classification (OR = 1.95 for T1/T2 vs. T3/T4, *p* = 0.005), and survival status (OR = 1.77 for alive vs. dead, *p* = 0.007).

### 3.4. Survival Outcomes of Liver Cancer Patients in the High and Low CEP55 Expression Groups

**Figure 4:**
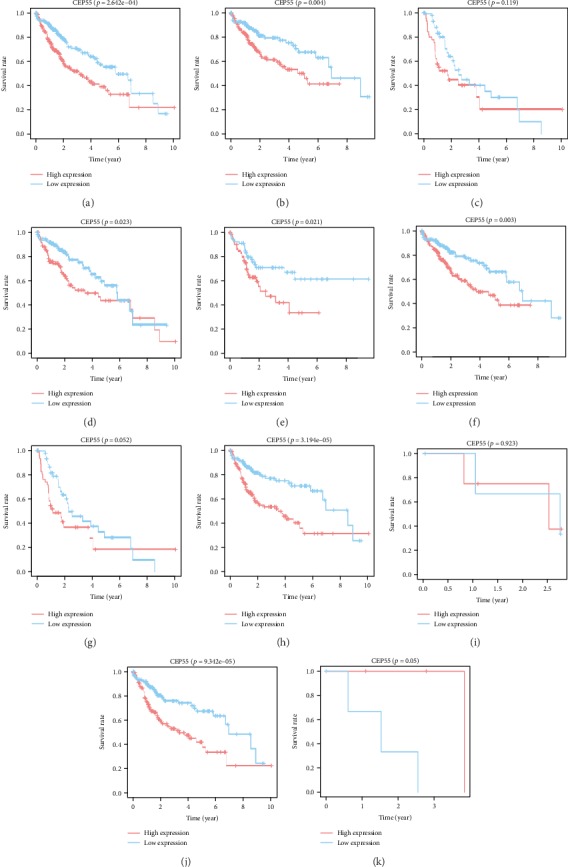
Survival analysis of all liver cancer patients (a) and subgroup analysis based on clinical stage (stage I/II and stage III/VI) (b, c), histological grade (G1/2 and G3/4) (d, e), and TNM classification (T1/2, T3/4, N0, N1, M0, M1) (f–k). Red curve indicates high CEP55 expression; blue curve indicates low CEP55 expression.

**Figure 5:**
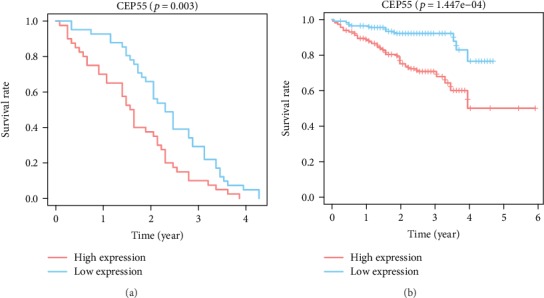
Survival analysis validation of CEP55. (a) Validation group of survival analysis in the GEO database (GSE54236). (b) Validation group of survival analysis in the ICGC database. GEO: Gene Expression Omnibus; ICGC: International Cancer Genome Consortium.

**Figure 6:**
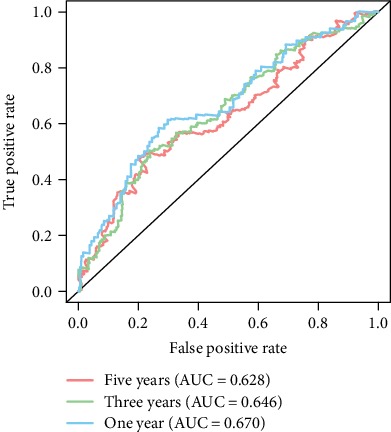
ROC curves of CEP55 for risk prediction in 1 year (blue curve), 3 years (green curve), and 5 years (red curve). ROC: receiver operating characteristics.

According to the Kaplan-Meier analysis for survival, patients having high CEP55 expressions were associated with dismal prognosis compared with those having low CEP55 expression (*p* = 2.642*e* − 4) (Figure 4(a)). Subgroup analysis showed that high CEP55 expression predominantly affected the prognosis of patients with clinical stage I/II, T1/T2, N0, and M0 disease (Figures 4(b), 4(c), and 4(f)–4(k)). However, whether patients are in grade G1/G2 or grade G3/G4, significant effects of high CEP55 expression to the prognosis can be detected (Figures 4(d) and 4(e)). Validations of survival analysis by GSE54236 and ICGC are shown in Figures 5(a) and 5(b). The area under the curve (AUC) of 1-, 3-, and 5-year ROC curves was 0.670, 0.646, and 0.628, respectively (Figure 6).

### 3.5. Risk Factors for OS in Liver Cancer Patients

**Figure 7:**
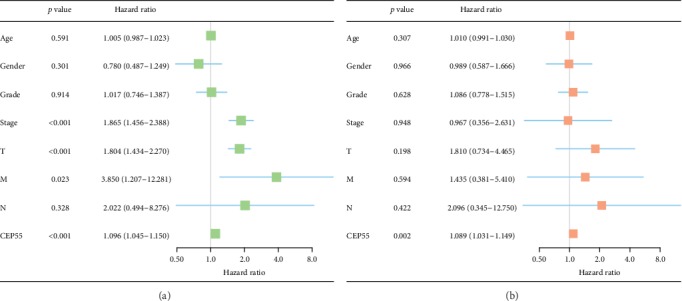
Univariate and multivariate analysis of the correlation between clinicopathological characteristics and OS. (a) Univariate analysis. (b) Multivariate analysis. OS: overall survival.

A total of 235 patients with complete data were selected and included in the multivariate and univariate analyses. Univariate analysis suggested that high CEP55 expression (HR = 1.10, 95% CI: 1.04-1.15, *p* = 1.79*e* − 4), advanced stage (HR = 1.86, 95% CI: 1.46-2.39, *p* = 8.07*e* − 7), high T classification (HR = 1.80, 95% CI: 1.43-2.27, *p* = 4.73*e* − 7), and distant metastasis (HR = 3.85, 95% CI: 1.21-12.28, *p* = 0.02) showed significant associations with poor survival. Upon multivariate analysis, the CEP55 level served as the risk factor to independently predict OS for patients with liver tumors (HR = 1.09, 95% CI: 1.03-1.15, *p* = 0.002) (Table 3).  Figure 7 presents the results of univariate and multivariate analysis illustrated by the forest plot.

### 3.6. GO and KEGG Analyses

**Figure 8:**
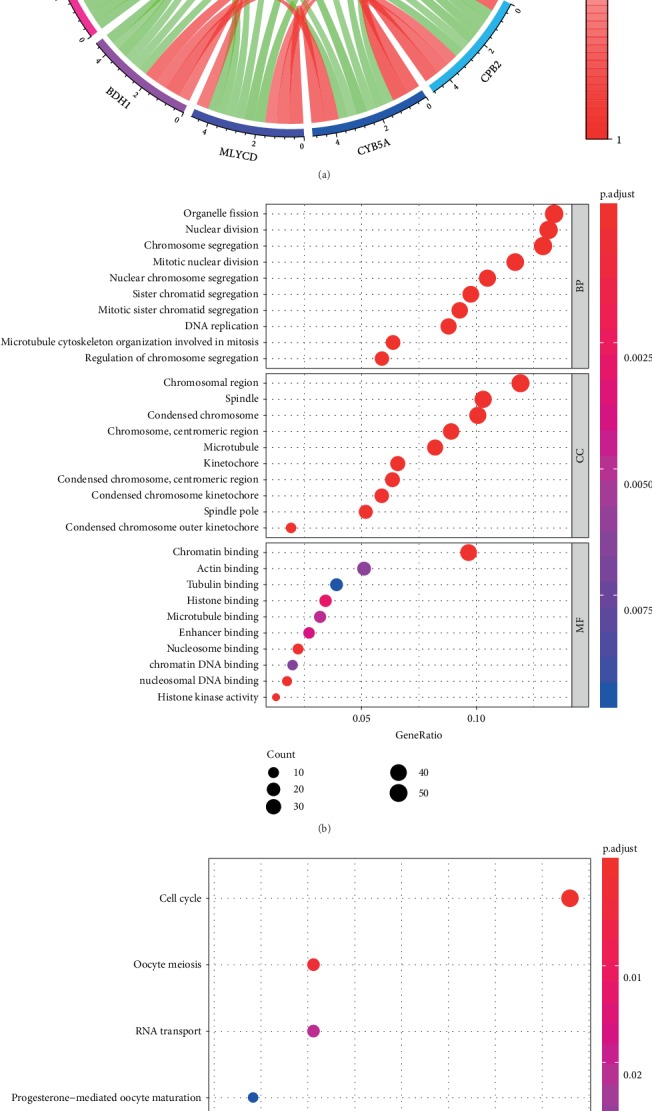
(a) The top 10 genes significantly correlated with CEP55 (red indicates positive correlation; green indicates negative correlation). (b) The top 10 significant terms of GO analysis (BP/CC/MF). (c) Six terms of KEGG analysis. GO: Gene Ontology; KEGG: Kyoto Encyclopedia of Genes and Genomes; BP: biological process; CC: cellular component; MF: molecular function.

A total of 458 genes were used for KEGG and GO enrichment analyses to explore the CEP55-related pathways and biological functions. The top 10 genes relevant to CEP55 are shown in Figure 8(a), among which five genes are positively correlated with CEP55 (KIF11, GTSE1, PLK1, LMNB2, ANLN) and another five genes are negatively correlated with CEP55 (2-Mar, CPB2, CYB5A, MLYCD, BDH1). The top 10 significant terms of GO enrichment analysis are presented in Figure 8(b), such as chromosomal region and spindle in the cellular component (CC), chromatin binding and actin binding in molecular function (MF), and organelle fission and nuclear division in the biological process (BP). A total of six KEGG pathways were identified, such as cell cycle, p53 signaling pathway, and DNA replication (Figure 8(c)).

### 3.7. Identification of CEP55-Related Signaling Pathways by GSEA

**Figure 9:**
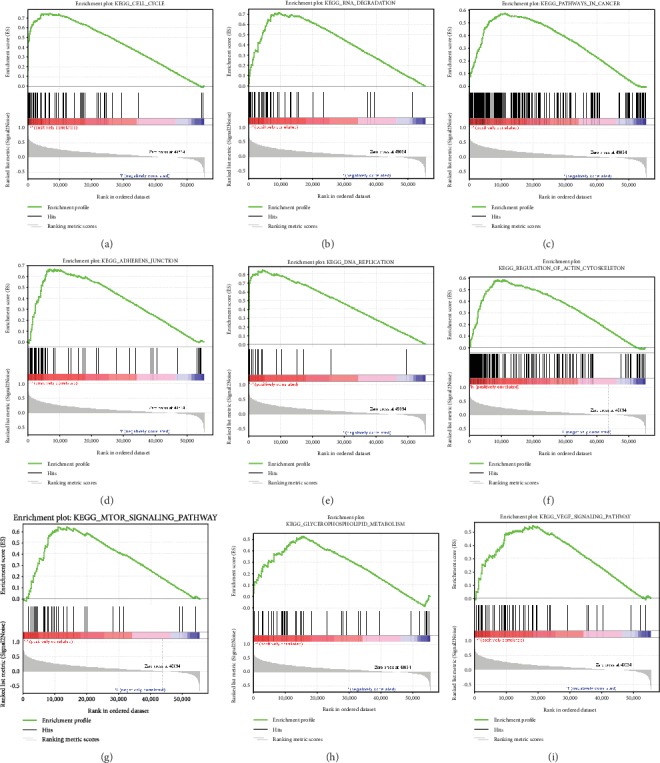
Enrichment plots from gene set enrichment analysis (GSEA). GSEA results showing cell cycle (a), RNA degradation (b), pathways in cancer (c), adherens junction (d), DNA replication (e), regulation of actin cytoskeleton (f), mTOR signaling pathway (g), glycerophospholipid metabolism (h), and VEGF signaling pathway (i) were differentially enriched in CEP55 high expression phenotype. NES: normalized ES; NOM *p* value: normalized *p* value; FDR: false discovery rate.

High and low CEP55 expression data were compared by GSEA for identifying the differentially activated signal transduction pathways within liver tumors. Results of GSEA revealed significant differences in MSigDB collection enrichment (c2.cp.kegg.v6.2.symbols.gmt). Gene sets related to the cell cycle, RNA degradation, pathways in cancer, the adherens junction, DNA replication, the mTOR signaling pathway, the regulation of actin cytoskeleton, glycerophospholipid metabolism, and the VEGF signaling pathway showed differential enrichment in the phenotype having high CEP55 level (Table 4 and Figures 9(a)–9(i)).

### 3.8. Correlation between TIICs and CEP55

**Figure 10:**
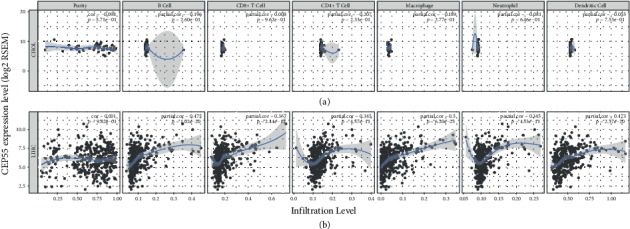
(a) The correlation between TIIC infiltration levels and CEP55 expression in CCA. (b) The correlation between TIIC infiltration levels and CEP55 expression in HCC. TIICs include B cells, CD4+ T cells, CD8+ T cells, neutrophils, macrophages, and dendritic cells. TIICs: tumor-infiltrating immune cells; CCA: cholangiocarcinoma; HCC: hepatocellular carcinoma.

Growing evidence showed that tumor immune environment is related to the prognosis in various cancers; we further explored the correlation between TIICs and CEP55. The results indicated that CEP55 expression was significantly related to the infiltration level of B cells (*R* = 0.475, *p* = 1.02*e* − 20), CD4+ T cells (*R* = 0.345, *p* = 4.57*e* − 11), CD8+ T cells (*R* = 0.367, *p* = 2.44*e* − 12), macrophages (*R* = 0.5, *p* = 6.20*e* − 23), neutrophils (*R* = 0.345, *p* = 4.53*e* − 13), and dendritic cells (*R* = 0.473, *p* = 2.37*e* − 20) in HCC (Figure 10(b)). However, no significant relationship was detected between CEP55 expression and the infiltration level of any TIICs in CCA (Figure 10(a)).

### 3.9. Prognostic Value of DNA Methylation of CEP55

**Figure 11:**
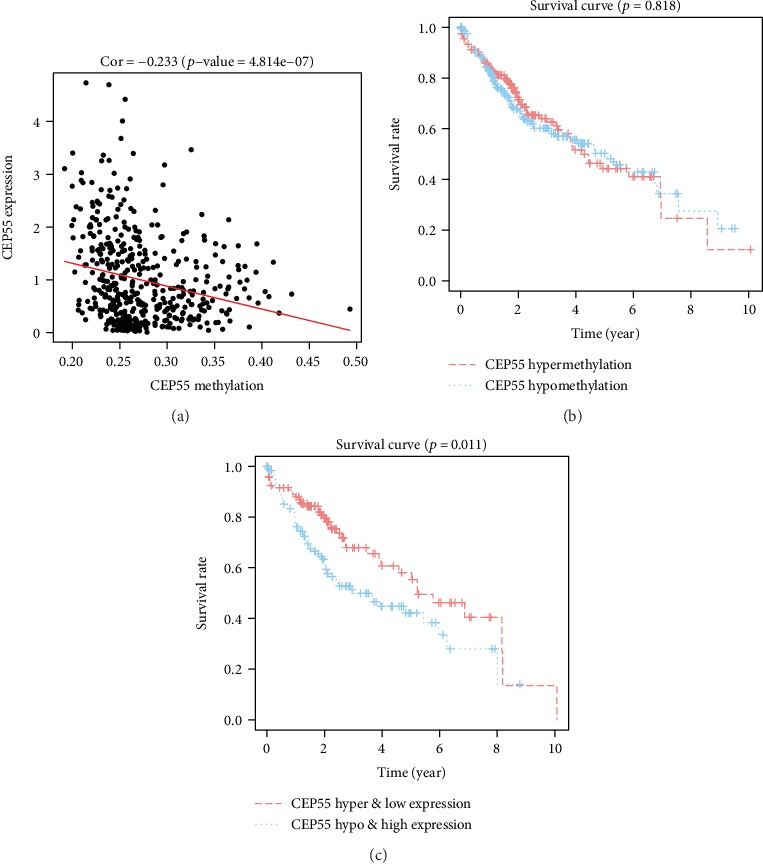
(a) Correlation between CEPP55 methylation and its mRNA expression. (b) Survival analysis based on CEP55 methylation. (c) Survival analysis based on the combination of methylation and mRNA expression of CEP55.

The result of the Pearson correlation analysis showed that CEP55 methylation level was negatively correlated to its mRNA expression (*R* = −0.233, *p* = 4.814*e* − 7) (Figure 11(a)). However, there was no significant difference of OS rates between the CEP55 hypermethylation and hypomethylation groups (*p* = 0.818) (Figure 11(b)). Therefore, we combined the methylation and the expression of CEP55 and classified liver cancer patients into the CEP55 hypermethylation and low expression group and the CEP55 hypomethylation and high expression group based on the median values. The Kaplan-Meier analysis was performed to compare the OS rates between the two groups, and the result indicated that the CEP55 hypermethylation and low expression group can achieve a better prognosis than the CEP55 hypomethylation and high expression group (*p* = 0.011) (Figure 11(c)).

## 4. Discussion

In this study, we conducted bioinformatic analysis using high-throughput RNA-sequencing data obtained from TCGA. The results showed that the expression of CEP55 in liver tumor tissues was significantly higher than that in adjacent normal tissues. CEP55 overexpression showed correlation with the dismal prognosis in liver cancer patients. In addition, high CEP55 expression in liver cancer was positively related to advanced stage, high histological grade, and high T classification. CEP55 expression had been recognized to be an independent risk factor in predicting liver cancer patient survival upon univariate and multivariate analyses.

CEP55, initially described as a midbody-related protein, has the size of 55 kDa, and it consists of 464 amino acids. It plays a critical role in regulating the physical cytokinesis [20]. CEP55 upregulation was previously reported to promote the migration and invasion of tumor cells and is related to the dismal prognosis for lung cancer [16], breast cancer [21], oral squamous cell carcinoma [22], cervical cancer [23], and osteosarcoma [24]. Such finding was consistent with our findings in liver cancer. The knockdown of CEP55 was found to inhibit tumor cell viability and proliferation [15, 25]. The overexpression of CEP55 can lead to disordered cytokinesis and an increase in multinucleated cells, which is an oncogenic feature in tumorigenesis [26]. According to the results in this study, CEP55 expression showed association with differentiation within liver tumor tissues. The higher the expression of CEP55 was, the poorer the differentiation of tumor cells. Similar results were found in prostate cancer and nasopharyngeal carcinoma [27, 28]. These findings clearly suggest the significance of CEP55 in tumorigenesis.

To further explore the role of CEP55 within liver tumors, enrichment analysis was performed and the results showed that cell cycle, DNA replication, pathways in cancer, mTOR signaling pathway, and VEGF signaling pathway were significantly enriched in the high CEP55 expression group. As a cell cycle and proliferation gene, the evidence of CEP55 overexpression promoting tumor progression in many cancers is reasonable. A recent study reported that mTOR can stimulate glycerophospholipid synthesis and that increased lipogenesis is associated with increased mTOR activity and the occurrence of HCC in humans [29]. Angiogenesis has been recognized as the essential condition for cancer growth, while VEGF has been identified as its critical regulator. VEGF was overexpressed within liver tumor, and VEGF level showed significant relationship with the clinical classification as well as lymph node and lung metastases [30]. CEP55 may promote liver tumor cell growth and metastasis through the VEGF signaling pathway.

Currently, surgical resection is the best method for the treatment of early liver cancer patients. Due to the difficulty in early diagnosis, most liver cancer patients, especially in China, are diagnosed at an advanced stage. Liver cancer is highly malignant, the therapeutic options for patients with advanced liver cancer are limited, and the therapeutic effect is poor. The 5-year liver cancer survival rate normalized by age is only 10.1% in China [31]. Therefore, we explored the association between CEP55 expression and OS. We found that CEP55 may be helpful in guiding treatment selection for liver cancer patients. Subgroup analysis results suggested that high CEP55 expression predominantly affected the prognosis of patients with clinical stage I/II, T1/T2, N0, and M0 disease, thus implying the special prognostic value of CEP55 and its potential contribution to the accurate treatment of liver cancer. However, few cases at advanced stage and high TNM classification may have led to this result, and future studies with large samples are needed to verify this conclusion.

TIICs are important parts of the tumor microenvironment, which are related to patient outcome and tumor behavior [32]. For the first time, we used the TIMER database to uncover the association between CEP55 expression and TIICs in liver cancer. Our results indicated that there is a significantly positive relationship between the CEP55 expression level and the infiltration level of B cells, CD4+ T cells, CD8+ T cells, macrophages, neutrophils, and dendritic cells in HCC. These correlations suggest that CEP55 may play an important role in the recruitment and regulation of immune cells in HCC. CEP55 is a tumor-associated antigen due to its aberrant expression in cancer. This property makes CEP55 an ideal candidate for cancer vaccine therapies. Some studies have reported the effectiveness of CEP55-based immunotherapy vaccines in the treatment of chemotherapy-resistant colon cancer stem cells and tumor-initiating cells [33, 34]. However, in vitro/vivo experiment are required to determine whether CEP55-based immunotherapy vaccines can be effectively used in HCC. In terms of CCA, no significant relationship was detected between CEP55 expression and the infiltration level of any TIICs. We speculate that this result may be due to the relatively small sample size of CCA.

DNA methylation is a common reason for gene expression change in tumors [35, 36]. In order to explore the potential mechanism of overexpression in liver cancer, we first used DNA methylation and gene expression data of liver cancer patients in the TCGA database to analyze the correlation between DNA methylation and gene expression of CEP55. We found that CEP55 expression is negatively correlated with its DNA methylation level, suggesting that DNA hypomethylation is one of the underlying causes for the overexpression of CEP55 in liver cancer. In addition, we performed survival analysis to investigate the prognostic value of the CEP55 methylation level in liver cancer patients. However, the results showed that the CEP55 methylation level did not affect the prognosis of liver cancer patients. Then, joint prognostic survival analysis was performed and we found that the combination of CEP55 methylation and its expression had a significant correlation with the prognosis of liver cancer patients. Patients with CEP55 hypermethylation and low expression can achieve a better prognosis than those with CEP55 hypomethylation and high expression. Therefore, we suppose that the CEP55 methylation level does not directly affect the prognosis of liver cancer patients, but by regulating the expression of CEP55 and then affecting the prognosis of liver cancer patients. Further study is needed to confirm this conjecture in the future.

The present study improves our understanding of the association between CEP55 and liver cancer, but some limitations still exist. First, this study was conducted based on the data obtained from a public database. Although we have made a validation of survival analysis and protein expression of CEP55 based on the data from the ICGC, GEO, and HPA databases, further experiments are needed to explore the molecular mechanisms associated with CEP55 in liver cancer progression and prognosis. Second, our evidence indicating the role of CEP55 as a prognostic predictor is limited to patients with early-stage disease and low TNM classification. It is essential to identify more effective predictors in advanced liver cancer patients in the future.

## 5. Conclusions

In summary, CEP55 is overexpressed in liver tumor tissues compared to normal liver tissues and CEP55 overexpression is related to dismal prognosis and increased immune infiltration levels of B cells, CD4+ T cells, CD8+ T cells, macrophages, neutrophils, and dendritic cells in liver cancer. DNA hypomethylation of CEP55 may contribute to its overexpression in liver cancer. CEP55 might serve as a candidate treatment target for liver cancer. Further basic or clinical experiments are needed to prove the biological impact of CEP55 in liver cancer.

## Figures and Tables

**Table 1:** Characteristics of liver cancer patients in the TCGA database.

Characteristics	Number of cases	Percentages (%)
Age		
<65	224	53.59
≥65	152	36.36
Not available	42	10.05
Gender		
Male	272	65.07
Female	146	34.93
Survival status		
Alive	271	64.83
Dead	147	35.17
Histological type		
HCC	377	90.19
CCA	41	9.81
Stage		
I	194	46.41
II	98	23.44
III	90	21.53
IV	12	2.87
Not available	24	5.75
Histological grade		
G1	55	13.16
G2	180	43.06
G3	124	29.67
G4	13	3.11
Not available	46	11.00
T classification		
T1	204	48.80
T2	107	25.60
T3	90	21.53
T4	14	3.35
Not available	3	0.72
N classification		
N0	290	69.38
N1	8	1.91
Not available	120	28.71
M classification		
M0	303	72.49
M1	8	1.91
Not available	107	25.60

Abbreviations: HCC: hepatocellular carcinoma; CCA: cholangiocarcinoma.

**Table 2:** CEP55 expression associated with clinical pathological characteristics (logistic regression).

Clinical characteristics	Total (*N*)	Odds ratio in CEP55 expression	*p* value
Age (≥65 vs.<65)	370	1.76 (1.16-2.69)	**0.008**
Gender (female vs. male)	404	0.64 (0.43-0.97)	**0.037**
Stage (I/II vs. III/IV)	380	1.97 (1.23-3.19)	**0.005**
Histological grade (G1/G2 vs. G3/G4)	366	3.06 (1.97-4.81)	**9.08** **e** **-7**
T (T1/T2 vs. T3/T4)	415	1.95 (1.23-3.12)	**0.005**
N (N0 vs. N1)	285	7.36 (1.29-138.56)	0.063
M (M0 vs. M1)	300	1.34 (029-0.91)	0.703
Survival status (alive vs. dead)	404	1.77 (1.17-2.68)	**0.007**
Histological type (HCC vs. CCA)	404	18.13 (5.38-112.99)	**8.43** **e** **-5**

Bold values indicate statistical significance. Abbreviations: HCC: hepatocellular carcinoma; CCA: cholangiocarcinoma.

**Table 3:** Univariate and multivariate analysis of the correlation between clinicopathological characteristics and OS in liver cancer patients.

Characteristics	Univariate analysis			Multivariate analysis		
	HR	95% CI	*p* value	HR	95% CI	*p* value
Age	1.01	0.99-1.02	0.59	1.01	0.99-1.03	0.31
Gender	0.78	0.49-1.25	0.30	0.99	0.59-1.67	0.97
Histologic grade	1.02	0.75-1.39	0.91	1.09	0.78-1.52	0.63
Stage	1.86	1.46-2.39	**8.07** **e** **-7**	0.97	0.36-2.63	0.95
T classification	1.80	1.43-2.27	**4.73** **e** **-7**	1.81	0.73-4.47	0.20
N classification	2.02	0.49-8.28	0.33	2.10	0.34-12.75	0.42
M classification	3.85	1.21-12.28	**0.02**	1.44	0.38-5.41	0.59
CEP55	1.10	1.04-1.15	**1.79** **e** **-4**	1.09	1.03-1.15	**0.002**

Bold values indicate statistical significance. Abbreviations: CI: confidence interval; HR: hazard ratio.

**Table 4:** Gene sets enriched in the high CEP55 expression phenotype.

Gene set name	NES	NOM *p*-val	FDR *q*-val
KEGG_CELL_CYCLE	2.10	<0.001	0.006
KEGG_RNA_DEGRADATION	2.04	<0.001	0.007
KEGG_PATHWAYS_IN_CANCER	1.98	<0.001	0.007
KEGG_ADHERENS_JUNCTION	1.95	<0.001	0.006
KEGG_DNA_REPLICATION	1.95	<0.001	0.006
KEGG_REGULATION_OF_ACTIN_CYTOSKELETON	1.93	<0.001	0.005
KEGG_MTOR_SIGNALING_PATHWAY	1.86	0.004	0.010
KEGG_GLYCEROPHOSPHOLIPID_METABOLISM	1.85	<0.001	0.011
KEGG_VEGF_SIGNALING_PATHWAY	1.82	<0.001	0.012

Abbreviations: NES: normalized enrichment score; NOM: normalized; FDR: false discovery rate.

## Data Availability

The data used to support the findings of this study are included within the article.

## Abbreviations

CEP55: Centrosome protein 55
TCGA: The Cancer Genome Atlas
GEO: Gene Expression Omnibus
ICGC: International Cancer Genome Consortium
TIICs: Tumor-infiltrating immune cells
TIMER: Tumor Immune Estimation Resource
OS: Overall survival
HCC: Hepatocellular carcinoma
CCA: Cholangiocarcinoma
HBV: Hepatitis b virus
IHC: Immunohistochemistry
HPA: The Human Protein Atlas
GO: Gene Ontology
KEGG: Kyoto Encyclopedia of Genes and Genomes
GSEA: Gene set enrichment analysis
ROC: Receiver operating characteristics
FDR: False discovery rate
NOM: Normalized
HR: Hazard ratio
OR: Odds ratio
CI: Confidence interval
CC: Cellular component
MF: Molecular function
BP: Biological process.

## References

[1] Villanueva A. (2019). Hepatocellular carcinoma. *The New England Journal of Medicine*.

[2] McGlynn K. A., Petrick J. L., London W. T. (2015). Global epidemiology of hepatocellular carcinoma: an emphasis on demographic and regional variability. *Clinics in Liver Disease*.

[3] Jemal A., Ward E. M., Johnson C. J. (2017). Annual report to the nation on the status of cancer, 1975-2014, featuring survival. *Journal of the National Cancer Institute*.

[4] Agromayor M., Martin-Serrano J. (2013). Knowing when to cut and run: mechanisms that control cytokinetic abscission. *Trends in Cell Biology*.

[5] Jeffery J., Sinha D., Srihari S., Kalimutho M., Khanna K. K. (2016). Beyond cytokinesis: the emerging roles of CEP55 in tumorigenesis. *Oncogene*.

[6] Lee H. H., Elia N., Ghirlando R., Lippincott-Schwartz J., Hurley J. H. (2008). Midbody targeting of the ESCRT machinery by a noncanonical coiled coil in CEP55. *Science*.

[7] Mondal G., Rowley M., Guidugli L., Wu J., Pankratz V. S., Couch F. J. (2012). BRCA2 localization to the midbody by filamin A regulates cep 55 signaling and completion of cytokinesis. *Developmental Cell*.

[8] Bastos R. N., Barr F. A. (2010). Plk 1 negatively regulates Cep 55 recruitment to the midbody to ensure orderly abscission. *The Journal of Cell Biology*.

[9] Inoda S., Hirohashi Y., Torigoe T. (2009). Cep55/c10orf3, a tumor antigen derived from a centrosome residing protein in breast carcinoma. *Journal of Immunotherapy*.

[10] Sinha D., Kalimutho M., Bowles J. (2018). Cep55 overexpression causes male-specific sterility in mice by suppressing Foxo1 nuclear retention through sustained activation of PI3K/Akt signaling. *The FASEB Journal*.

[11] Fratta E., Coral S., Covre A. (2011). The biology of cancer testis antigens: putative function, regulation and therapeutic potential. *Molecular Oncology*.

[12] Hwang C. F., Shiu L. Y., Su L. J. (2013). Oncogenic fibulin-5 promotes nasopharyngeal carcinoma cell metastasis through the FLJ10540/AKT pathway and correlates with poor prognosis. *PLoS One*.

[13] Chen C. H., Lai J. M., Chou T. Y. (2009). VEGFA upregulates FLJ10540 and modulates migration and invasion of lung cancer via PI3K/AKT pathway. *PLoS One*.

[14] Colak D., Nofal A., AlBakheet A. B. (2013). Age-specific gene expression signatures for breast tumors and cross-species conserved potential cancer progression markers in young women. *PLoS One*.

[15] Tao J., Zhi X., Tian Y. (2014). CEP55 contributes to human gastric carcinoma by regulating cell proliferation. *Tumour Biology*.

[16] Liu L., Mei Q., Zhao J., Dai Y., Fu Q. (2016). Suppression of CEP55 reduces cell viability and induces apoptosis in human lung cancer. *Oncology Reports*.

[17] Sinha D., Duijf P., Khanna K. K. (2019). Mitotic slippage: an old tale with a new twist. *Cell Cycle*.

[18] Li M., Gao J., Li D., Yin Y. (2018). CEP55 promotes cell motility via JAK2(-)STAT3(-)MMPs cascade in hepatocellular carcinoma. *Cells*.

[19] Li T., Fan J., Wang B. (2017). TIMER: a web server for comprehensive analysis of tumor-infiltrating immune cells. *Cancer Research*.

[20] Fabbro M., Zhou B. B., Takahashi M. (2005). Cdk1/Erk2- and Plk1-dependent phosphorylation of a centrosome protein, Cep55, is required for its recruitment to midbody and cytokinesis. *Developmental Cell*.

[21] Kalimutho M., Sinha D., Jeffery J. (2018). CEP55 is a determinant of cell fate during perturbed mitosis in breast cancer. *EMBO Molecular Medicine*.

[22] Chen C. H., Chien C. Y., Huang C. C. (2009). Expression of FLJ10540 is correlated with aggressiveness of oral cavity squamous cell carcinoma by stimulating cell migration and invasion through increased FOXM1 and MMP-2 activity. *Oncogene*.

[23] Qi J., Liu G., Wang F. (2018). High levels of centrosomal protein 55 expression is associated with poor clinical prognosis in patients with cervical cancer. *Oncology Letters*.

[24] Xu L., Xia C., Sheng F., Sun Q., Xiong J., Wang S. (2018). CEP55 promotes the proliferation and invasion of tumour cells via the AKT signalling pathway in osteosarcoma. *Carcinogenesis*.

[25] Wang Y., Jin T., Dai X., Xu J. (2016). Lentivirus-mediated knockdown of CEP55 suppresses cell proliferation of breast cancer cells. *Bioscience Trends*.

[26] Xu Z. Y., Ma X. S., Qi S. T. (2015). Cep55 regulates spindle organization and cell cycle progression in meiotic oocyte. *Scientific Reports*.

[27] Kulkarni P., Uversky V. N. (2017). Cancer/testis antigens: "smart" biomarkers for diagnosis and prognosis of prostate and other cancers. *International Journal of Molecular Sciences*.

[28] Chen C. H., Shiu L. Y., Su L. J. (2012). FLJ10540 is associated with tumor progression in nasopharyngeal carcinomas and contributes to nasopharyngeal cell proliferation, and metastasis via osteopontin/CD44 pathway. *Journal of Translational Medicine*.

[29] Guri Y., Colombi M., Dazert E. (2017). mTORC2 promotes tumorigenesis via lipid synthesis. *Cancer Cell*.

[30] Guo L. Y., Zhu P., Jin X. P. (2016). Association between the expression of HIF-1*α* and VEGF and prognostic implications in primary liver cancer. *Genetics and Molecular Research*.

[31] Zeng H., Zheng R., Guo Y. (2015). Cancer survival in China, 2003-2005: a population-based study. *International Journal of Cancer*.

[32] Cariani E., Pilli M., Zerbini A. (2012). Immunological and molecular correlates of disease recurrence after liver resection for hepatocellular carcinoma. *PLoS One*.

[33] Inoda S., Hirohashi Y., Torigoe T. (2011). Cytotoxic T lymphocytes efficiently recognize human colon cancer stem-like cells. *The American Journal of Pathology*.

[34] Inoda S., Morita R., Hirohashi Y. (2011). The feasibility of Cep55/c10orf3 derived peptide vaccine therapy for colorectal carcinoma. *Experimental and Molecular Pathology*.

[35] Esteller M. (2007). Cancer epigenomics: DNA methylomes and histone-modification maps. *Nature Reviews Genetics*.

[36] Baylin S. B., Ohm J. E. (2006). Epigenetic gene silencing in cancer - a mechanism for early oncogenic pathway addiction?. *Nature Reviews Cancer*.

